# A Novel Hierarchical Vision Transformer and Wavelet Time–Frequency Based on Multi-Source Information Fusion for Intelligent Fault Diagnosis

**DOI:** 10.3390/s24061799

**Published:** 2024-03-11

**Authors:** Changfen Gong, Rongrong Peng

**Affiliations:** School of Education, Nanchang Institute of Science and Technology, Nanchang 330108, China; prr19871102@126.com

**Keywords:** mechanical components, multi-source information fusion, DL, fault diagnosis, NHVT

## Abstract

Deep learning (DL) has been widely used to promote the development of intelligent fault diagnosis, bringing significant performance improvement. However, most of the existing methods cannot capture the temporal information and global features of mechanical equipment to collect sufficient fault information, resulting in performance collapse. Meanwhile, due to the complex and harsh operating environment, it is difficult to extract fault features stably and extensively using single-source fault diagnosis methods. Therefore, a novel hierarchical vision transformer (NHVT) and wavelet time–frequency architecture combined with a multi-source information fusion (MSIF) strategy has been suggested in this paper to boost stable performance by extracting and integrating rich features. The goal is to improve the end-to-end fault diagnosis performance of mechanical components. First, multi-source signals are transformed into two-dimensional time and frequency diagrams. Then, a novel hierarchical vision transformer is introduced to improve the nonlinear representation of feature maps to enrich fault features. Next, multi-source information diagrams are fused into the proposed NHVT to produce more comprehensive presentations. Finally, we employed two different multi-source datasets to verify the superiority of the proposed NHVT. Then, NHVT outperformed the state-of-the-art approach (SOTA) on the multi-source dataset of mechanical components, and the experimental results show that it is able to extract useful features from multi-source information.

## 1. Introduction

Mechanical components (i.e., bearings and gears) are increasingly important in many heavy and oversized engineering fields (e.g., oil production, mining, and construction) as a vital part of modern industrial society. Stable and efficient operation of mechanical equipment is an essential prerequisite for economic progress and the security of life [1,2]. However, failures of mechanical components represent the great majority of mechanical equipment failures. To assure the continuous, reliable, safe, and efficient functioning of mechanical equipment and to promote the stable advancement of the manufacturing industry, it is necessary to conduct research on sophisticated and effective mechanical equipment fault diagnosis techniques [3,4,5].

The traditional machine learning (ML) methods mainly rely on expert knowledge and prior experience to select signal processing techniques, and then use manual thresholds for fault feature extraction and identification [6,7,8]. However, the diagnostic performance of traditional diagnostic models cannot meet industrial demands due to limitations such as human factors and the inability to handle large amounts of data. With the advent of DL and Industry 4.0, more researchers are turning to intelligent diagnosis methods for extracting, selecting, and classifying fault features in mechanical equipment. The prevailing DL fault diagnosis methods mainly include convolutional neural networks (CNNs), deep belief networks (DBNs), autoencoders (AEs), graph neural networks (GNNs), long short-term memory (LSTM), etc. For example, Shao et al. proposed unsupervised domain-share CNNs (UDSCNNs) to achieve fault transfer diagnosis under time-varying speeds [9]. Tang et al. constructed the novel adaptive CNNs to implement fault diagnosis by acoustic images [10]. Zhang et al. adopted the salp swarm algorithm to optimize the parameters of the DBNs for identifying bearing faults [11]. Wang et al. designed extended DBNs to exploit useful information and detect defects in the chemical process [12]. Yang et al. combined the improved sparse AEs and multilevel denoising strategy to achieve early fault diagnosis [13]. Liu et al. used GNNs to extract information from the constructed spatial–temporal diagrams to obtain the diagnosis of rotating machinery [14]. Wang et al. designed the novel BERT-BiLSTM-CRF model to extract information from the created fault knowledge graphs from the electric power equipment [15]. In particular, CNNs have gained more attention and recognition for their superior performance in diagnosing mechanical equipment failure through convolution and pooling operations. However, due to the interference of various noises in the real working environment, the periodic characteristics of multi-source signals are likely to be masked, leading to difficulties in extracting useful local features by CNNs. Additionally, the local nature of convolutional kernels makes it challenging to capture the context of multi-source signals, leading to a shortage of adequate fault representation [16]. To address the limitations imposed by the local receptive field, some scholars implement the global mining of fault information by combining recurrent neural networks. However, these methods can make the model structure more complex and lower diagnostic performance. The increase in the number of parameters not only increases the training time cost but also leads to overfitting [17,18,19]. To thoroughly address the limitations of the methods above, a new DL method called the Transformer [20] has been proposed to capture context-related features using the self-attention mechanism in the token space. As a result, the Transformer has encountered a new use in fault diagnosis research. Ding et al. combined an improved time–frequency Transformer and self-attention mechanism to extract fault abstractions from vibration signals [21]. Shao et al. designed an end-to-end Convformer-NSE framework to diagnose faults of gearboxes by fusing general and detail abstractions beneath intense noise [22]. Du et al. combined the denoising AEs and the Transformer to capture valuable and rich features for diagnosing mechanical equipment [23]. Therefore, the global property of the Transformer can enable it to obtain global characteristics to collect sufficient fault information and improve fault diagnosis performance.

The background of most mechanical equipment fault diagnosis methods is based on experimental environments since collecting original data from a single sensor or a signal source is sufficient to obtain satisfactory diagnostic performance. However, these research results fail to achieve good performance under actual operating conditions of mechanical equipment because they require attention to three key issues: (1) Poor anti-interference capability. In real operating conditions, irregular noise can overlap with the signal or data where the fault features are located, making it more difficult to extract and mine the fault features, thereby reducing fault diagnosis accuracy. (2) Poor generalization capability. More minor changes in operating conditions may lead to algorithm failure or reduced accuracy. (3) Poor feature extraction capability. Complex real environments may have multiple sources of interference and noise, which can affect sensor performance and accuracy, resulting in localized information reflecting only specific locations or conditions [24]. Regarding the above issues, multi-information fusion technology is gradually applied to the research of fault diagnosis for intelligent monitoring of critical components in mechanical equipment. For instance, Ribeiro et al. proposed multi-head 1D CNNs to handle multi-source sensor data to increase feature extraction and achieve real faults of the electric motors [25]. Yang et al. constructed multi-channel graphs through multi-sensor data and then used improved GCNs for rotating machinery diagnosis [26]. Zhang et al. introduced an improved AdaBoost algorithm to fuse vibration and acoustic signals to obtain the fault diagnosis findings [27]. Li et al. offered an adaptive multi-source information fusion strategy to describe the health status of mechanical equipment [28]. Chen et al. employed multiple DL methods to process original signals to obtain multi-source information for gear fault diagnosis [29]. Xie et al. transformed multi-source sensor data into RGB images and then adopted the improved CNN and the residual network to validate the operating conditions of the mechanical equipment [30]. Hence, the multi-source fusion method can be an excellent solution to the problems faced by traditional DL methods. In essence, the multi-source information fusion method realizes the association, crossover, and complementarity of multiple information sources to make the fault feature extraction more comprehensive and improve the detection, characterization, and identification of faults by the model [31].

In this research, we take ideas from the Transformer and existing multi-source information fusion approaches and then apply them to create a new framework for monitoring and diagnosing health states of mechanical equipment by using the NHVT to learn more valuable fault abstractions from multi-sensor information fusion. In the proposed NHVT, to fuse data from multiple sources, we employ a time–frequency method to map one-dimensional signals from various sources onto a time–frequency representation. Finally, our proposed framework can simultaneously extract both comprehensive and discriminative abstractions from multi-sensor information by combining NHVT and multi-sensor information and produces more stable and accurate diagnostic results compared to SOTA methods when dealing with different diagnostic tasks (i.e., different proportions of training samples). The primary ideas of this paper can be summed up as follows:

(1) The novel hierarchical vision Transformer is proposed to enable end-to-end diagnosis of the critical component in mechanical equipment by modeling the multi-source information in a united deep network.
(2) The WT is used to transform the original signals to the time–frequency for rich and comprehensive fault features. Then, the data-level fusion strategy is proposed to form the input data for retaining more fault-based information.
(3) The novel SwinTransformer framework is established to realize fault diagnosis in extracting context information of multi-source information under different training sample ratios, including realization formulas and corresponding loss functions.
(4) Comprehensive tests are run on two multi-source information datasets of the mechanical equipment to demonstrate the superior performance of the proposed NHVT. In addition, the key parameters and noise resistance in the proposed NHVT are discussed to provide interpretability.

This paper is divided into the following sections: The fundamental knowledge and theoretical framework are presented in Section 2. Then, the core procedure of the proposed method is described in depth in Section 3. Two case studies and the diagnostic performance of the proposed method across a variety of diagnosis tasks are presented in Section 4, along with the descriptions of the multi-source information experimental platforms. The subsequent explanation of this proposed methodology is detailed in Section 5. Section 6 concludes this paper and discusses directions for further research.

## 2. Theoretical Background

### 2.1. Wavelet Time–Frequency Transform

Wavelet time–frequency transform, also known as wavelet transform (WT), is a mathematical technique to analyze signals in both the time and frequency domains [32,33]. Unlike the classical Fast Fourier transform (FFT), which provides a fixed frequency resolution throughout the signal, WT can provide variable time and frequency resolution, making it suitable for analyzing non-stationary signals.

The core idea of the WT is to decompose the target signal into a set of wavelet functions, including the so-called mother wavelet function and its dilated and translational functions. Most importantly, it can provide information about the energy distribution of the signal across time and frequency scales, revealing details about its transient behavior, frequency content, and time-localized features. Hence, WT can offer a powerful tool for analyzing non-stationary signals and capturing both temporal and spectral characteristics simultaneously, making it a valuable technique in many scientific and engineering applications.

### 2.2. Multi-Source Information Fusion Strategy

Multi-source information fusion combines information from multiple sources or sensors to obtain more accurate, robust, and comprehensive representations [34,35]. Researchers develop the idea of multi-source information fusion to get beyond the shortcomings of data from a single source or a single sensor. Each source or sensor may provide partial, noisy, or incomplete information, but combining them makes it possible to improve the overall quality of the information and make wiser decisions [36].

**Figure 1:**
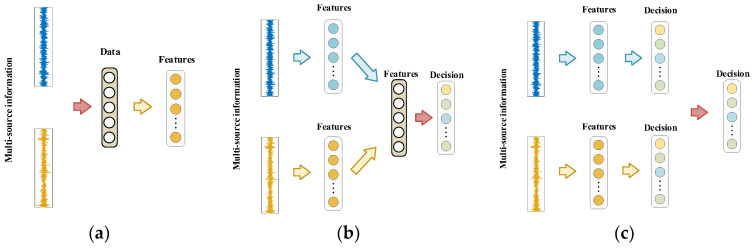
Multi-source information fusion methods. (**a**) Data-level fusion; (**b**) Feature-level fusion; (**c**) Decision-level fusion.

There are three distinct categories of multi-source information fusion strategy:

a. Data-level fusion: This involves combining data from various sources directly to save all useful information, as represented in Figure 1a.
b. Feature-level fusion: This involves combining features extracted from different sources or sensors to create a unified feature representation, as shown in Figure 1b.
c. Decision-level fusion: This involves decisions or predictions made by individual sources or sensors to make a final decision, as displayed in Figure 1c.

### 2.3. Transformer

**Figure 2:**
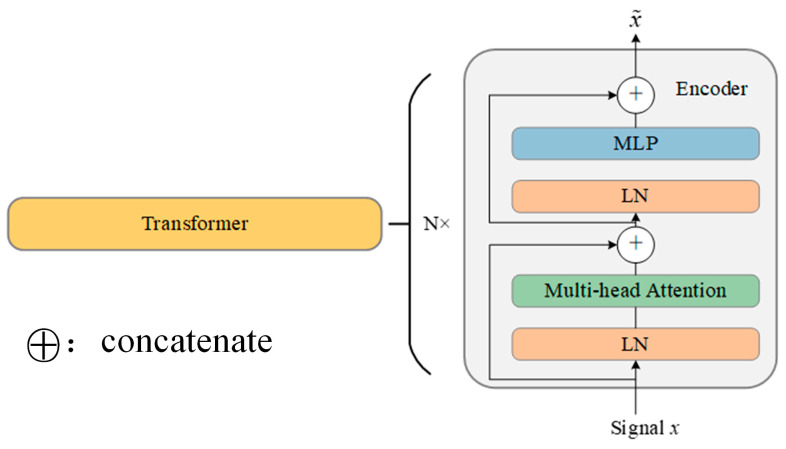
Basic structure of Transformer.

The Transformer architecture can enable the model to measure different parts of the input data differently, depending on their correlation with the corresponding task. The traditional Transformer consists of several encoders and decoders with the same structure. The proposed method can extract the compressed representation information from the original signals using a stack of *N* identical encoders, as shown in Figure 2.

As displayed in Figure 2, the Transformer mainly includes the multi-head self-attention module for the former and the multi-layer perceptron module for the latter. In addition, the layer normalization technique (LN) is implemented both before and after the multi-head self-attention module to reduce the likelihood of gradient explosion and disappearance and further improve the precision and training efficacy of the proposed method. Then, the residual connections are incorporated into the Transformer to achieve higher performance. Hence, the training process of the Transformer can be written as

(1) $$x^{\prime }=x+Multihead(LN(x))$$

where *x* denotes the input of the Transformer.

## 3. Proposed Method

### 3.1. SwinTransformer

**Figure 3:**
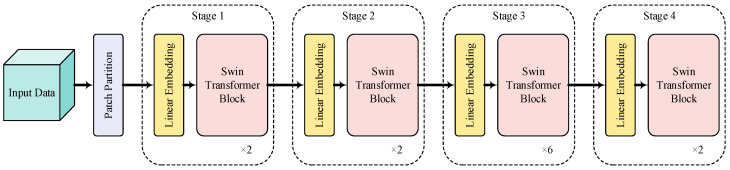
Overview of the SwinTransformer architecture.

The traditional Transformer needs to compute the relationships between all tokens to create global adaptation. However, it tends to produce significant computational complexity. Hence, the SwinTransformer is adopted in this paper to address this issue and boost the performance of this proposed method. Figure 3 shows the overview of the SwinTransformer architecture [37]. The patch partition module splits input WT diagrams of vibration and acoustic signals to several non-overlapping patches. Then, these patches are applied by modified self-attention computation (i.e., SwinTransformer modules). Hence, the Linear embedding module and SwinTransformer module are known as Stage 1. The regular and shifted windowing multi-head self-attention modules (R-MSA and SW-MSA, respectively) are adopted for more effective modeling. Given the dimension of the input data m×n, the computational complexity can be written as follows:

(2) $$\Omega (R-\mathrm{MSA})=4hwC^{2}+2{\left(hw\right)}^{2}C$$

(3) $$\Omega (\mathrm{SW}-\mathrm{MSA})=4hwC^{2}+2M^{2}hwC$$

where *C* denotes the output dimension; *h* and *w* mean the height and width of the input data; and *M* is the constant.

To further generate rich feature representations, the patch merging module can be used to decrease the number of tokens as the network layers of the proposed NHVT deepen. The role of the patch merging module is to concatenate the features of the adjacent patches and then output deeper elements through the linear layer. Next, the SwinTransformer module is applied after the patch merging module for further feature extraction and transformation, referred to as Stage 2. At the same time, the above process is repeated twice, referred to as Stage 3 and Stage 4, respectively. By stacking these processes, hierarchical features are extracted for fault diagnosis of mechanical equipment.

**Figure 4:**
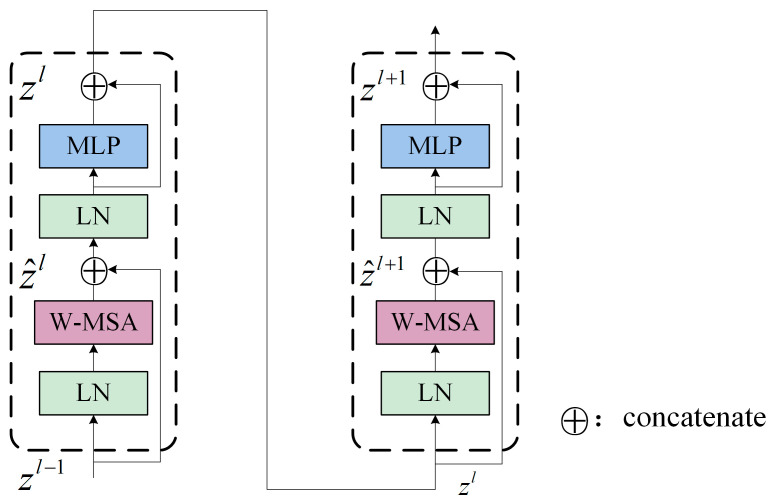
Two successive SwinTransformer blocks.

Meanwhile, the shifted window partitioning strategy is applied to the successive SwinTransformer modules to further enhance the feature mining performance of the proposed NHVT, as shown in Figure 4.

(4) $${\hat{z}}^{l}=R-\mathrm{MSA}\left(\mathrm{LN}\left(z^{l-1}\right)\right)+z^{l-1}$$

(5) $$z^{l}=\mathrm{MLP}\left(\mathrm{LN}\left({\hat{z}}^{l}\right)\right)+{\hat{z}}^{l}$$

(6) $${\hat{z}}^{l+1}=\mathrm{SW}-\mathrm{MSA}\left(\mathrm{LN}\left(z^{l}\right)\right)+z^{l}$$

(7) $$z^{l+1}=\mathrm{MLP}\left(\mathrm{LN}\left({\hat{z}}^{l+1}\right)\right)+{\hat{z}}^{l+1}$$

where ${\hat{z}}^{l}$ means the output of the R-MSA module; ${\hat{z}}^{l+1}$ denotes the outcome of the SW-MSA module; and $z^{l}$ and $z^{l+1}$ represent the output of the linear layer. The shift window partitioning approach can provide links between neighboring non-overlapping windows in the preceding layer, which can be proven effective in fault diagnosis.

### 3.2. Multi-Source Information Fusion Strategy

**Figure 5:**
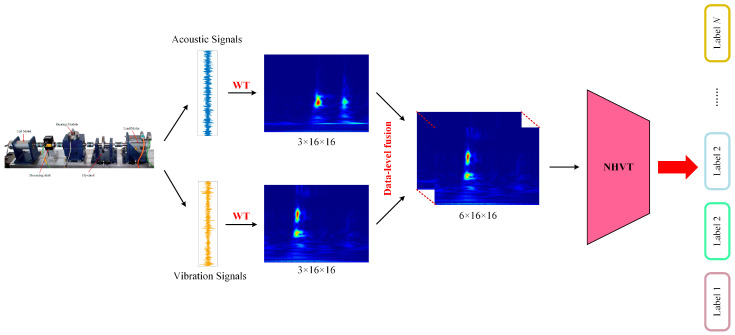
Multi-source information data-level fusion strategy.

In this proposed NHVT, we use the data-level method strategy to retain the original information from multi-source data without significant loss or modification. This allows for the direct combination of raw or pre-processed data, ensuring every crucial detail is noticed and discarded. This can be particularly advantageous when the specific features or characteristics of the individual data sources are essential for the analysis. Most importantly, the data-level fusion method offers advantages in preserving information, enhancing feature representation, reducing dimensionality, facilitating early integration, improving robustness, and simplifying the analysis pipeline. Specifically, the multi-source information data-level fusion strategy of the NHVT is described in Figure 5.

### 3.3. Overall Framework

**Figure 6:**
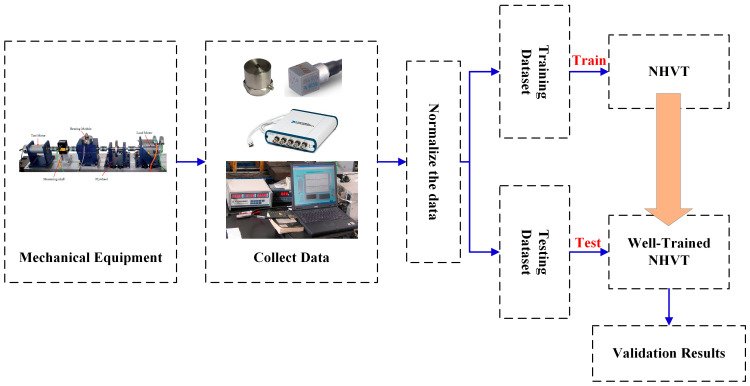
Overall framework of the proposed method.

This section introduces the overview procedure of the proposed NHVT for identifying the health conditions of mechanical components in the mechanical equipment under multi-source information datasets. Figure 6 provides a detailed explanation of the overall structure, followed by a rundown of the individual steps.

(1) Step I: Collect multi-source original signals (i.e., vibration signals, current signals, and acoustic signals) from mechanical equipment experimental rigs.
(2) Step II: Standardize the gathered multi-source original data and add white Gaussian noise with different signal-to-noise ratios in the Section 5.
(3) Step III: Transform the normalized multi-source data into WT diagrams, randomly sample time–frequency diagrams, and finally, partition them into training and testing datasets.
(4) Step IV: Implement the multi-source training samples in two case studies with different proportions to train the proposed NHVT.
(5) Step V: Use the multi-source testing samples in two case studies to validate the diagnostic performance of the proposed method.

## 4. Case Validation

In this section, two multi-source datasets are used to train and test the proposed method to detect and diagnose faults. Meanwhile, several SOTA models are also provided for the sake of comparison to further demonstrate the precision and efficiency of the proposed NHVT.

### 4.1. Case Study I: Fault Diagnosis of Paderborn Multi-Source Information Dataset

#### 4.1.1. Multi-Source Information Dataset Overview

**Figure 7:**
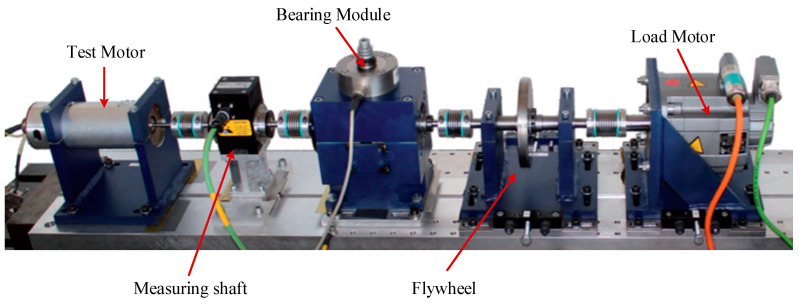
Schematic diagram of the lab bench.

The Paderborn University introduces the Paderborn multi-source information dataset to evaluate fault diagnosis of bearings, and then the laboratory bench is introduced in Figure 7.

**Figure 8:**
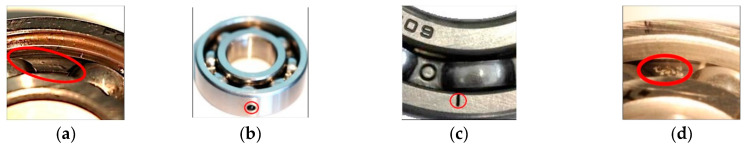
The diagram of the laboratory bench. (**a**) Fatigue pitting; (**b**) Drilling holes; (**c**) Electrical discharge trenches; (**d**) Electric engraver pitting.

The multi-source information in this case study means the vibration and current signals, where the piezoelectric accelerometer and current sensor acquire them. Meanwhile, the sampling frequency of multi-source sensors is set at 64,000 Hz. This bearing dataset contains measurements of four bearings, each of which experienced different types and levels of damage involving inner race, outer race, and ball defects, as seen in Figure 8. The bearings were run at various speeds and loads, and the multi-source signals were recorded and collected using sensors mounted on the bearings.

It is worth noting that the operating conditions in this case study include rotational speed (1000 Rpm), load (0.7 Nm), and force (1000 N). A detailed description of the dataset is listed in Table 1.

**Figure 9:**
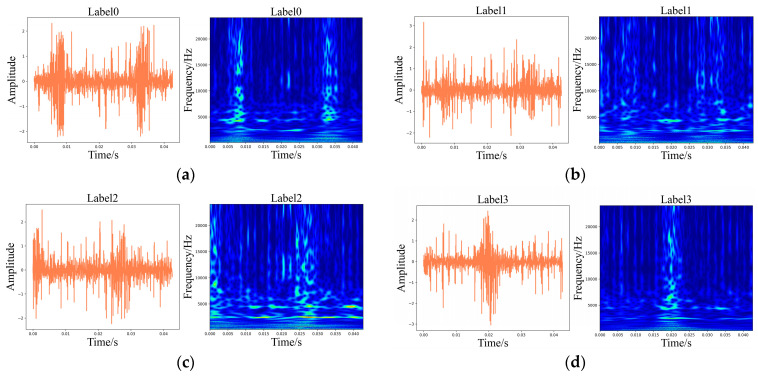

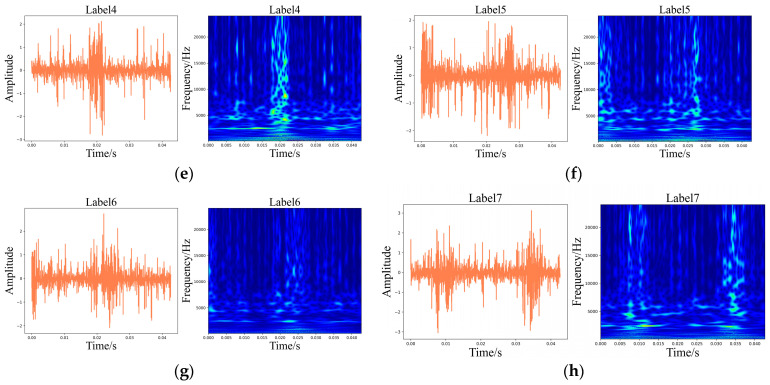
Time-domain illustrations of the vibration signals and corresponding WT diagrams. (**a**) Label 0; (**b**) Label 1; (**c**) Label 2; (**d**) Label 3; (**e**) Label 4; (**f**) Label 5; (**g**) Label 6; (**h**) Label 7.

**Figure 10:**
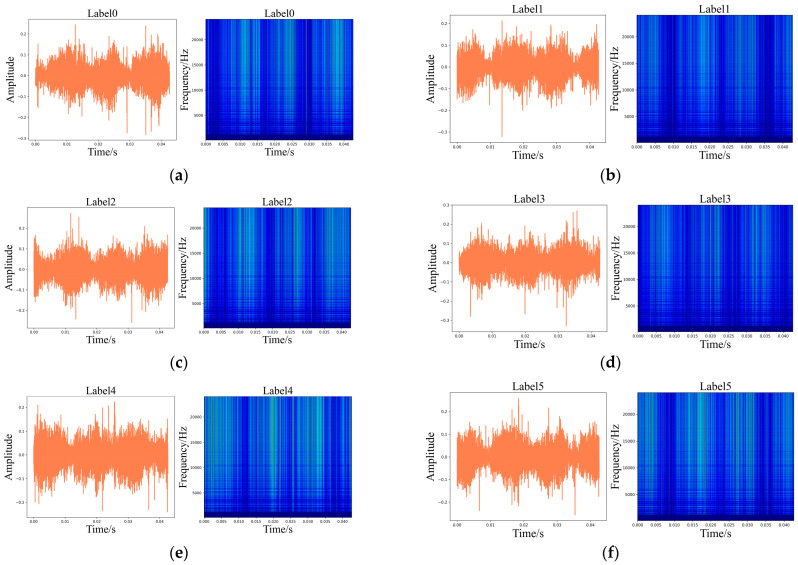

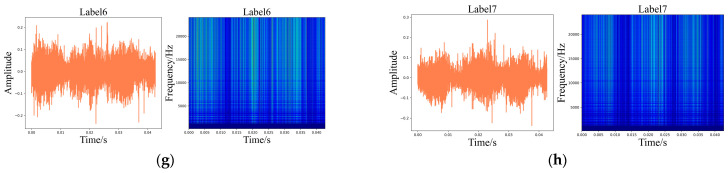
Time-domain illustrations of the current signals and corresponding WT diagrams. (**a**) Label 0; (**b**) Label 1; (**c**) Label 2; (**d**) Label 3; (**e**) Label 4; (**f**) Label 5; (**g**) Label 6; (**h**) Label 7.

Meanwhile, Figure 9 and Figure 10 display the time-domain and WT illustrations of the vibration and current signals, respectively.

#### 4.1.2. Experimental Details

Meanwhile, four different faulty types in different locations of the bearings (i.e., fatigue pitting, drilling holes, electrical discharge trenches, and electric engraver pitting) are designed so that each health state is viewed as the working condition. The gathered vibration and current signals are standardized through the utilization of the Z-score normalization technique. Subsequently, the standard multi-source signals are partitioned into sub-samples using a 2048 window size. There is a total of 500 samples available for every health state. Ninety percent are selected randomly for the training dataset, while the rest are used for the testing dataset. In conclusion, there are 3600 multi-source information samples in the training dataset and 400 multi-source information samples in the testing dataset.

#### 4.1.3. Comparison Methods and Implementation Details

To illustrate the advantages of fault diagnosis in the proposed NHVT, the STOA methods have been implemented on identical diagnostic cases to achieve fair comparisons.

Single-source information methods:

(1) CNNs. The basic method without a multi-source information strategy.
(2) DBNs. The basic method without a multi-source information strategy.
(3) SAEs. The basic method without a multi-source information strategy.

Multi-source information methods:

(4) SDPVGG. By combining multi-source information symmetry dot pattern and a Visual Geometry Group 16 network, the decision-level fusion strategy is employed to achieve fault diagnosis of mechanical components [38].
(5) MH1DCNNs. These employ the multi-head 1D CNNs to extract valuable features from multi-source original signals for practical motor fault diagnosis [25].
(6) 2DCNN-Adaboost. This uses the improved 2DCNNs and a novel Adaboost with a dynamic deletion mechanism to achieve more comprehensive fault diagnosis of bearings [27].
(7) MSICNNs. By using the improved 1DCNNs and 2DCNNs, the multi-source sensing information can be fused to achieve the health status of the rolling mill [39].
(8) MSIDBNs. These embed the improved single-sensor DBNs into the framework to extract the rich and complementary multi-source information from multi-source signals [40].

The initial weights are randomly selected, resulting in different initial states of the network at the beginning of each training. Consequently, this phenomenon may lead to different diagnostic results. To reduce the effects of randomness, it is noted that each procedure is executed ten times, and then the number of iteration epochs is set to 100. All methods are intended to have a learning rate of 0.001. The Adam optimization algorithm was then used to minimize the cross-entropy loss function, which was used during training. The Adam optimizer shows powerful generalization capability, and it is suitable for numerous diagnostic tasks in different case studies, which are more applicable to real engineering environments [41]. Meanwhile, the Adam optimizer is less affected by the learning rate; thus, it can acquire the optimal result during the training process [42,43]. Finally, these methods are realized using the 1.8.0 Pytorch and 3.8.13 Python Framework, tested on an AMD Ryzen 5800H with a GeForce RTX Nvidia 3060 GPU.

#### 4.1.4. Diagnosis Results

**Figure 11:**
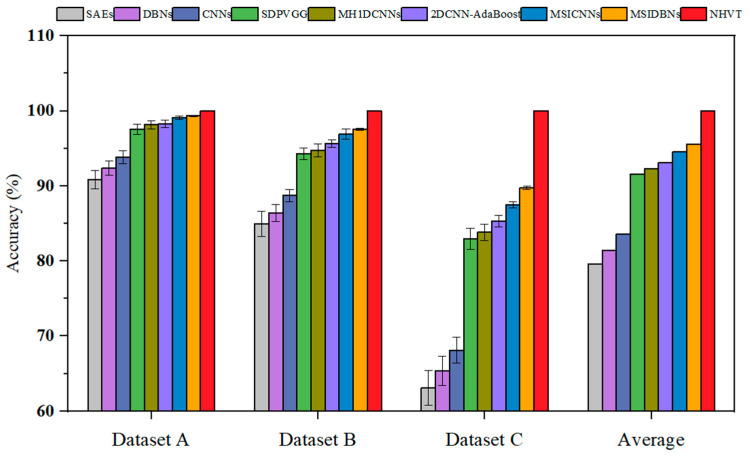
Fault diagnosis accuracy of all the methods.

**Figure 12:**
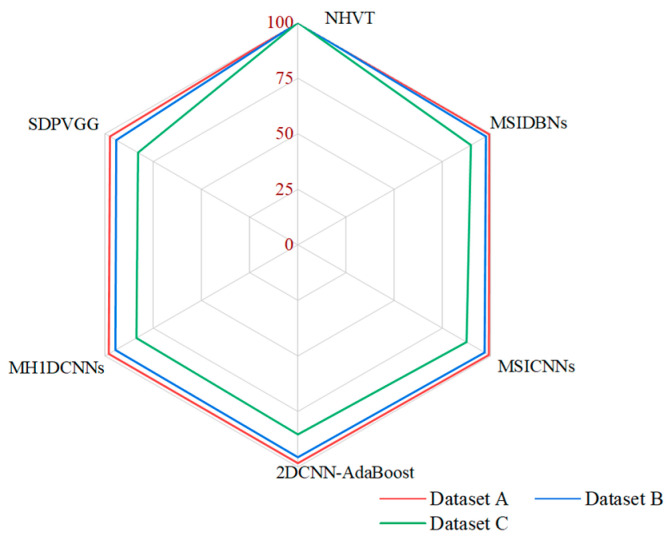
Radar chart of the average diagnostic results.

It was compared to the SOTA methods through various indicators to further demonstrate the strength and competency of the proposed NHVT. The diagnostic histogram of all the SOTA methods and the radar chart of the average diagnostic results with multi-source information fusion methods are displayed in Table 2 and Figure 11 and Figure 12, respectively.

Table 2 shows that all the methods obtain the optimal diagnosis results under Dataset A because of sufficient training data. Significantly, as the complexity of the task increases (i.e., the amount of training data decreases), the diagnostic performance of the proposed NHVT is still being approved compared to other methods because of robust nonlinear feature extraction capability. In detail, the proposed method improves by 0.68% and 0.91% compared to the best-performing method (i.e., MSIDBNs) and the second best-performing method (i.e., MSICNNs) in Dataset A. Then, the NHVT can still achieve 100% accuracy in Dataset B, which is significantly better than other methods. Next, the proposed NHVT can obtain the best accuracy among all the methods in Dataset C. Most importantly, all the multi-source information fusion methods (i.e., SDPVGG, MH1DCNNs, 2DCNN-AdaBoost, MSICNNs, MSIDBNs, NHVT) outperform the single-source information methods (i.e., SAEs, DBNs, CNNs) because the former methods enable access to the more comprehensive fault representations of mechanical equipment components. In conclusion, our proposed NHVT has the best diagnostic accuracy and the minor standard deviation among the three datasets.

**Figure 13:**
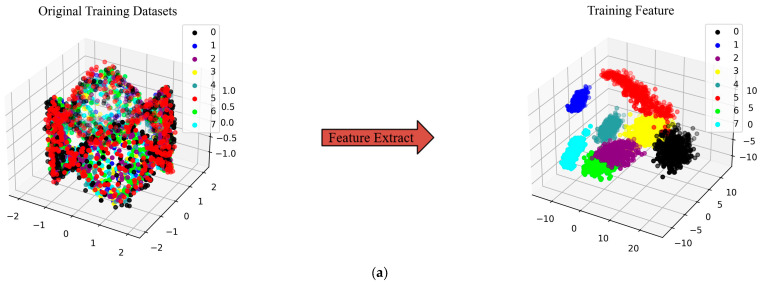

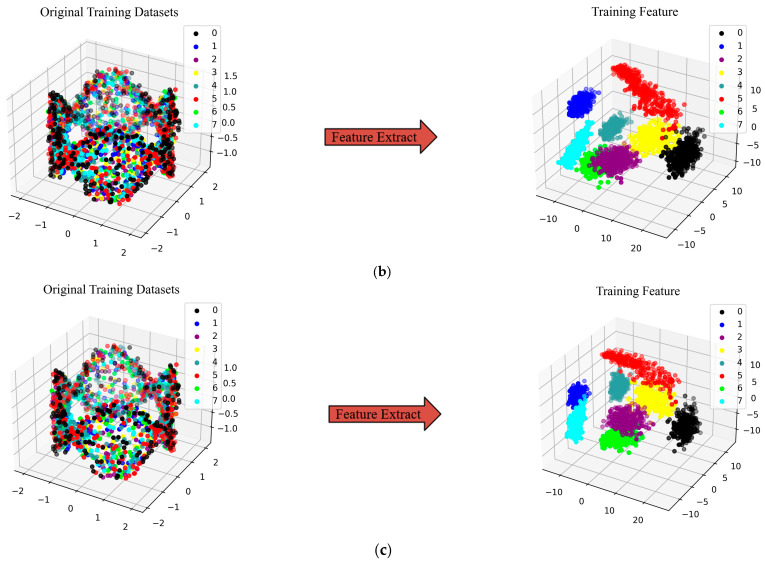
Feature visualization via the t-SNE of the proposed method. (**a**) Dataset A; (**b**) Dataset B; (**c**) Dataset C.

To showcase the effectiveness of fault diagnosis in the proposed NHVT and to directly experience the advantages of a multi-source information fusion strategy in the feature extraction process, t-Distributed Stochastic Neighbor Embedding (t-SNE) is employed to present features taken from the final hidden layer of the proposed method, as illustrated in Figure 13.

As shown in the left half of Figure 13, the two-dimensional features based on the multi-source signals are not well aligned. On the contrary, the two-dimensional features based on the final features are well divided into eight parts. Most importantly, the proposed method can cluster samples of the same category under different datasets. Then, features of the different categories are nicely differentiated, which indicates the higher quality of the extracted features of the proposed method.

**Figure 14:**
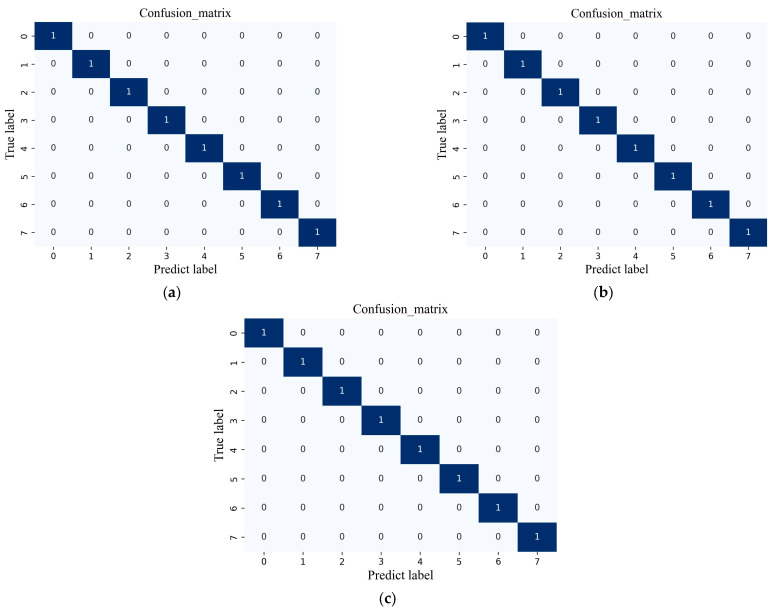
Confusion matrix of the proposed method. (**a**) Dataset A; (**b**) Dataset B; (**c**) Dataset C.

Meanwhile, the confusion matrix is utilized to assess the diagnosis effect of a classification model by comparing the predicted and actual labels of three datasets. This is a technique for summarizing the performance of a classification algorithm. The horizontal axis represents the predicted label, while the vertical axis represents the actual label. Figure 14 describes the results of the proposed NHVT through the confusion matrix in Dataset A, Dataset B, and Dataset C, respectively.

Based on its powerful feature extraction capability, the messages contained in the individual confusion matrices for recognizing eight health conditions of the mechanical equipment in three datasets are accurate. As a result, the proposed NHVT is shown to have satisfactory diagnostic accuracy (each condition reaches 100%).

**Figure 15:**
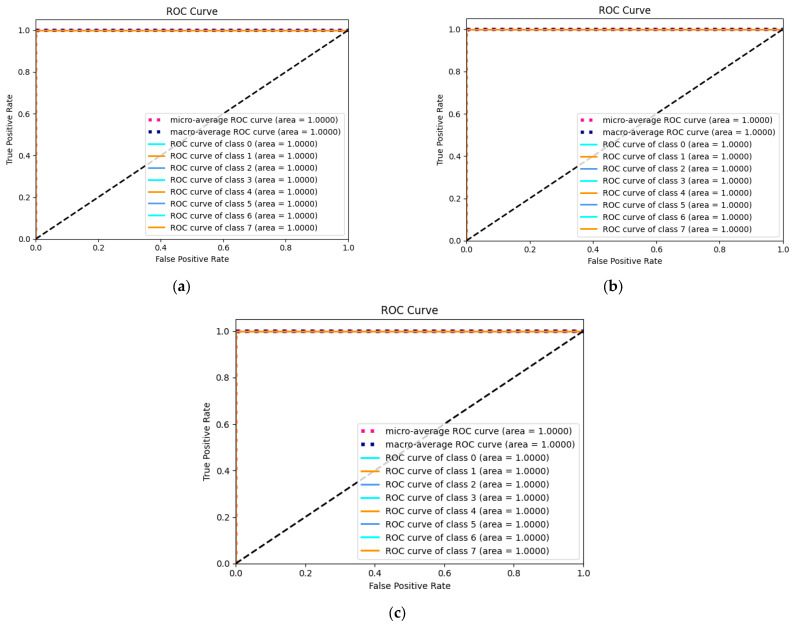
ROC curve of the proposed method. (**a**) Dataset A; (**b**) Dataset B; (**c**) Dataset C.

Finally, the Receiver Operating Characteristic (ROC) plotted by the actual positive rate (TPR) on the *y*-axis against the false positive rate (FPR) on the *x*-axis is used to illustrate the efficacy of the proposed NHVT to discriminate between positive and negative instances across various threshold settings visually, as shown in Figure 15.

As shown in Figure 15, the micro-average and macro-average ROC curves for all three datasets reach 100%. Meanwhile, all categories in all three datasets have an area under the curve of 1. Therefore, the proposed NHVT shows positive characteristics with three datasets, including low false positive levels, high flexibility, and poor wrong classification.

### 4.2. Case Study II: Fault Diagnosis of Cylindrical Multi-Source Information Dataset

#### 4.2.1. Multi-Source Information Dataset Overview

**Figure 16:**
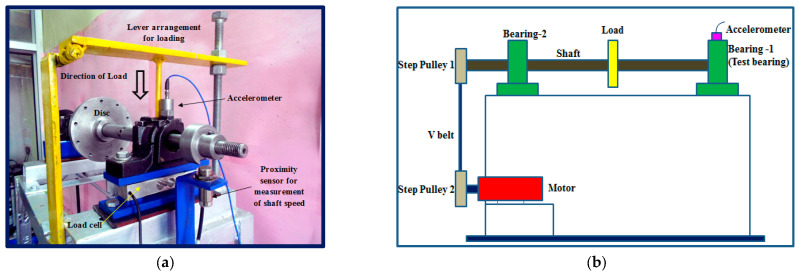
Cylindrical roller bearing test rig. (**a**) Actual working environment; (**b**) Schematic diagram.

The Mechanical Engineering Department develops the cylindrical test rig to diagnose the roller bearings, and then multi-source information containing vibration and acoustic signals is collected by the specialized data acquisition device. Figure 16a,b introduce the actual working situation and schematic diagram of the test rig, which mainly includes a motor, two pulleys, a load, and a test-bearing housing.

To record the vibration and acoustic signals of the bearings in varying health states, a triaxial accelerometer and an acoustic emission sensor are mounted on the top of the bearing housing and then sample data at a rate of 70,000 Hz. The fault diagnosis dataset consists of two working conditions and three fault types with different defect widths generated by an electrical discharge machining process.

**Figure 17:**
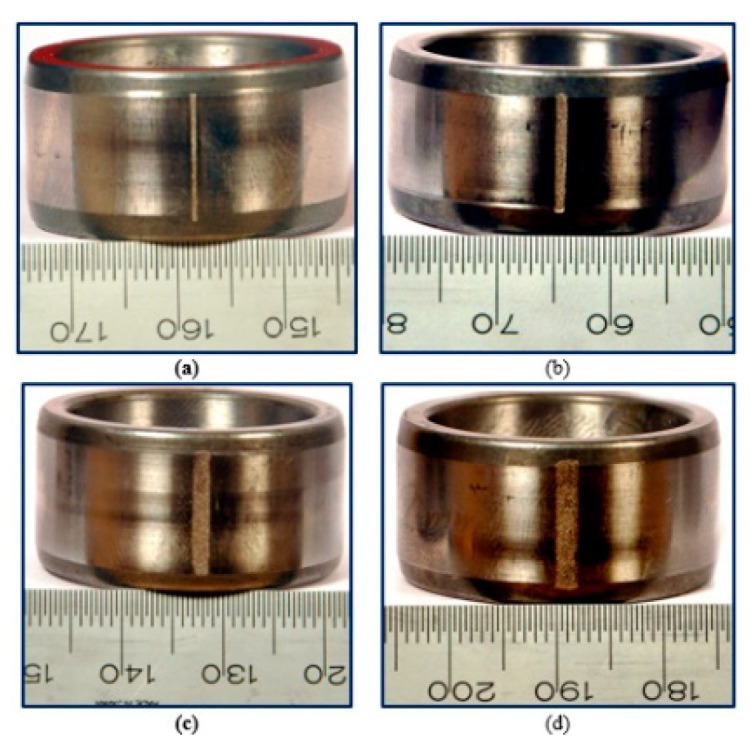
Fault width of inner race: (**a**) 0.43; (**b**) 1.01; (**c**) 1.56; (**d**) 2.03.

**Figure 18:**
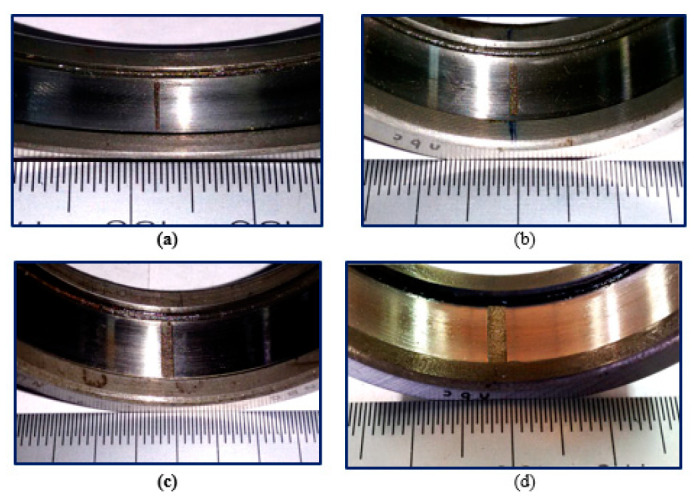
Fault width of outer race: (**a**) 0.42; (**b**) 1.16; (**c**) 1.73; (**d**) 2.12.

**Figure 19:**
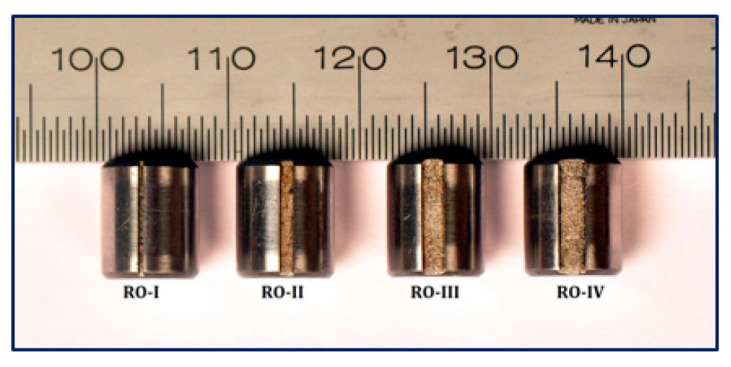
Fault width of roller.

The dataset is outlined in great depth in Table 3, and then the typical faulty forms with different defect widths in this case study can be seen in Figure 17, Figure 18 and Figure 19. The defective sizes of various fault types and the allocation strategies for the different datasets are described in detail.

**Figure 20:**
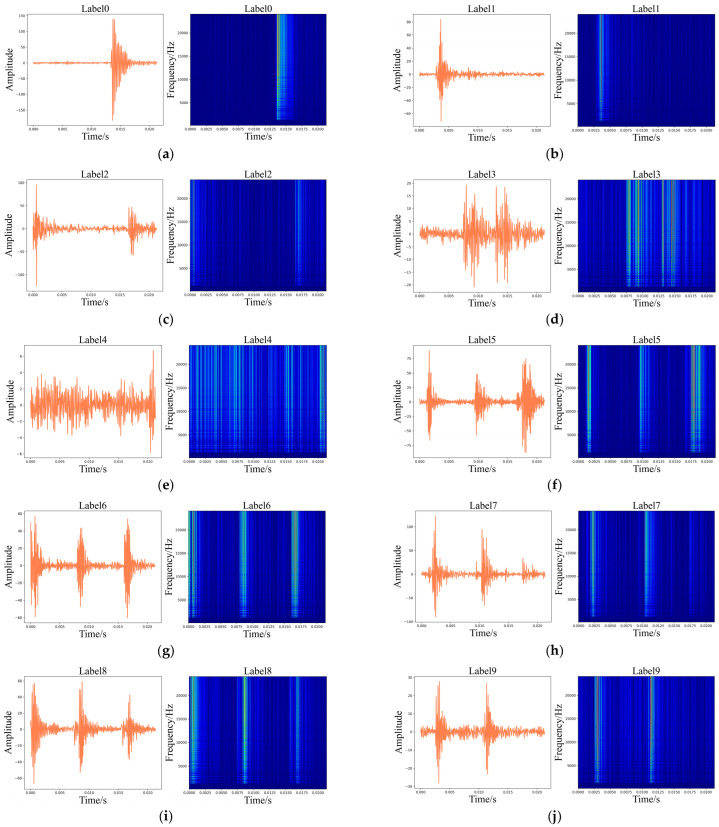

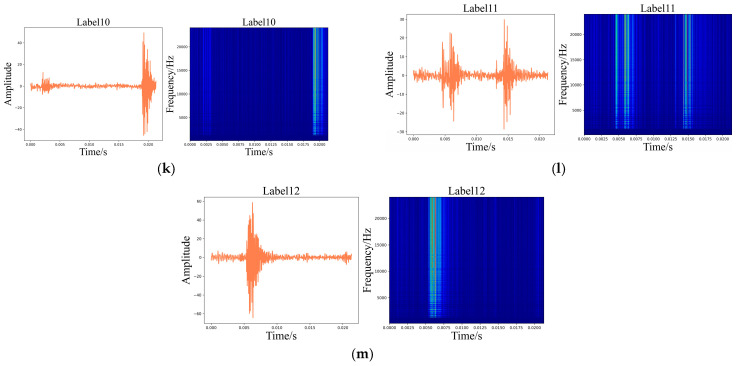
Time-domain diagrams of the vibration signals and corresponding WT diagrams. (**a**) Label 0; (**b**) Label 1; (**c**) Label 2; (**d**) Label 3; (**e**) Label 4; (**f**) Label 5; (**g**) Label 6; (**h**) Label 7; (**i**) Label 8; (**j**) Label 9; (**k**) Label 10; (**l**) Label 11; (**m**) Label 12.

**Figure 21:**
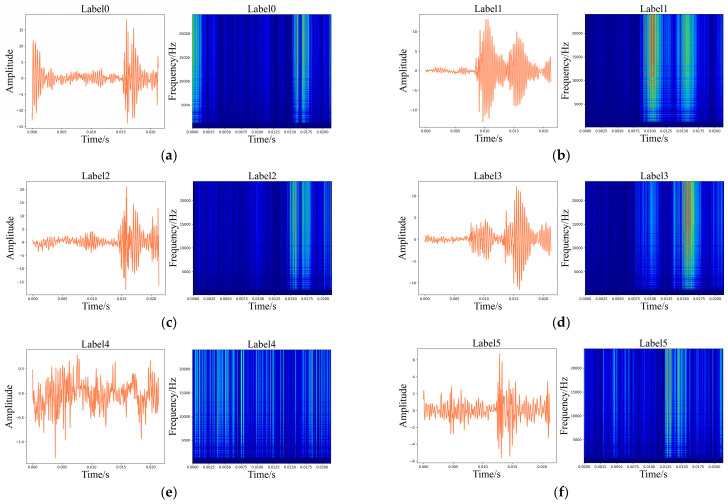

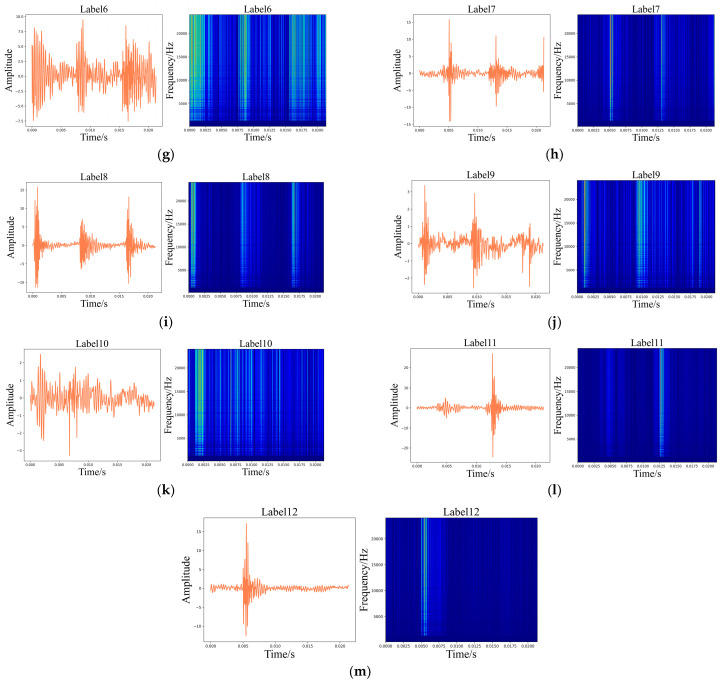
Time-domain diagrams of the acoustic signals and corresponding WT diagrams. (**a**) Label 0; (**b**) Label 1; (**c**) Label 2; (**d**) Label 3; (**e**) Label 4; (**f**) Label 5; (**g**) Label 6; (**h**) Label 7; (**i**) Label 8; (**j**) Label 9; (**k**) Label 10; (**l**) Label 11; (**m**) Label 12.

Meanwhile, the time-domain plots and corresponding WT graphics of acoustic signals and the diagrams are displayed in Figure 20 and Figure 21, respectively.

#### 4.2.2. Experimental Details

For the collected vibration and current signals, they are normalized through the use of the Z-score normalization technique. Subsequently, the standard multi-source signals are divided into sub-samples using a window size of 1024. Each health condition has a total of 100 samples available. Among them, 90% of the samples are randomly selected as the training dataset, and the remaining samples are allocated to the test dataset. Finally, the training dataset consists of 1170 samples, while the test dataset includes 130 samples.

To reduce the impact of randomness, each method is subjected to 10 repetitions, and then the number of iteration epochs is set to 100. All the methods are designed to have a learning rate of 0.001. The Adam optimization algorithm was then applied to the cross-entropy loss function during the training process to minimize it.

#### 4.2.3. Diagnosis Results

**Figure 22:**
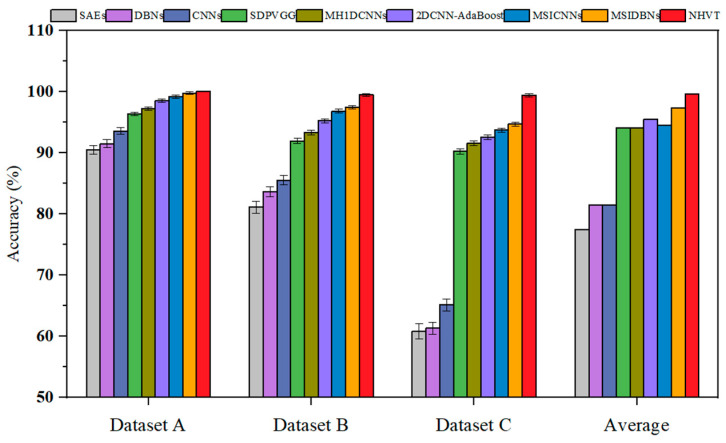
Fault diagnosis accuracy of all the methods.

The proposed NHVT was developed for the purpose of higher performance of fault diagnosis via multi-sensor information fusion. It was compared to the above SOTA models to further establish its superiority. Table 4 and Figure 22 show the standard experimental results and histogram distributions of these methods in this case study.

Figure 22 summarizes the diagnosis results and shows that all methods can achieve their best diagnostic effect and lowest standard deviation in Dataset A with a significant enough training dataset. Compared to the worst-performing method (SAEs) in Dataset C, the proposed method has a diagnostic accuracy of 99.38%. The diagnostic accuracy and stability of the multi-source information fusion methods are enhanced in comparison to the single-source information methods across three diagnosis tasks. Then, the diagnostic performance of the NHVT can be improved by 4.66%, 5.57%, 6.84%, and 7.81% when compared to that achieved by using multi-source information methods in Dataset C. Especially, the average diagnostic performance of multi-source information fusion methods is better than those of single-source information methods. It is worth noting that the proposed method can still manage to outperform SOTA methods on the most challenging task possible (Dataset C).

**Figure 23:**
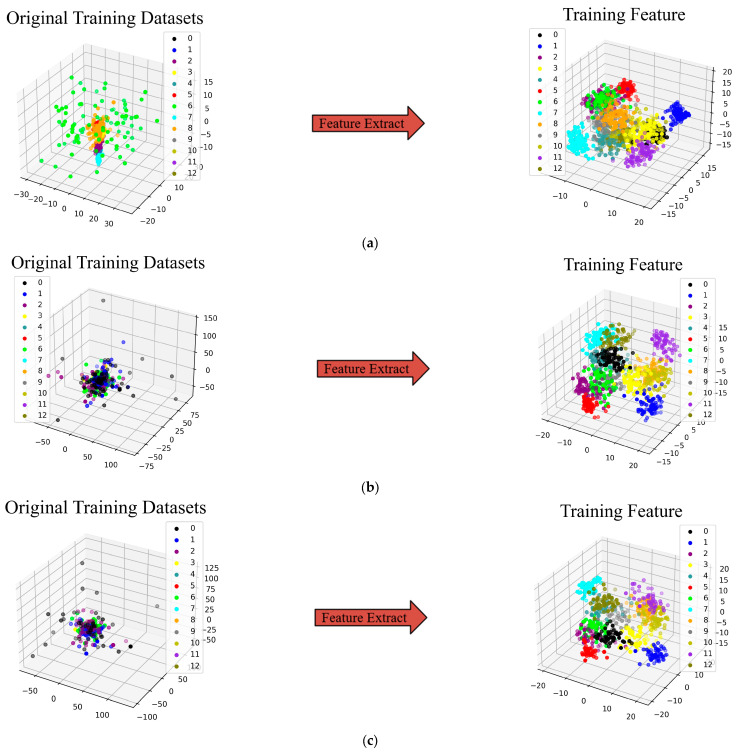
Feature visualization via the t-SNE of the proposed NHVT. (**a**) Dataset A; (**b**) Dataset B; (**c**) Dataset C.

The t-SNE technique is utilized to display the features obtained from the final hidden layer of the proposed method, demonstrating the superior performance of the proposed NHVT and then allowing for an intuitive evaluation of the feature extraction ability based on multi-source information fusion, as illustrated in Figure 23.

It is clear that the original multi-source data represented by the t-SNE technique cannot differentiate between the various health conditions on the left half of the diagrams. In contrast, the health categories of the mechanical components may be easily detected after visualizing the features retrieved from the proposed NHVT. In summary, the t-SNE is a dimensionality reduction technique used for visualizing high-dimensional multi-source data that can help us better understand the structure and patterns of the multi-source information. By adjusting parameters and understanding the t-SNE technique, we can correctly appreciate its results and then apply the proposed NHVT to diagnose the health states. It can be observed from the above figure that the proposed method exhibits higher separability between categories, even in the face of the most challenging diagnostic task (Dataset C). Therefore, the proposed method can effectively extract valuable features from multi-source information.

**Figure 24:**
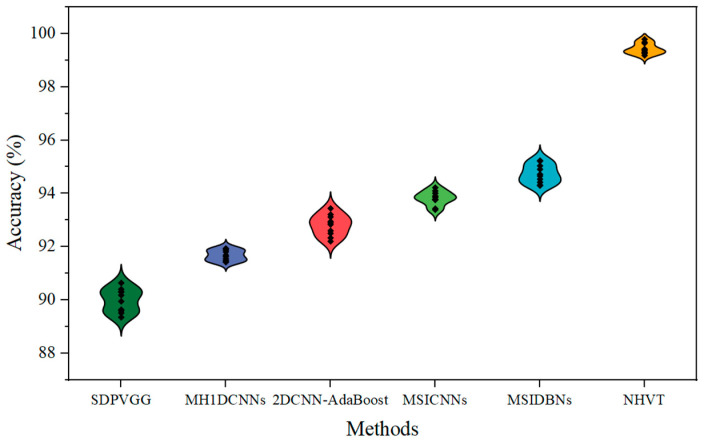
Violin plot and scatter plot of multi-source information fusion methods.

After that, to demonstrate the stability of the proposed NHVT, the diagnostic results of multi-source information fusion methods based on ten experiments in Dataset C of Case II are described in the form of violin and scatter diagrams in Figure 24.

Therefore, as shown in Figure 24, the proposed NHVT can not only achieve a more centralized and reliable data distribution, but it can also have the maximum classification performance.

**Figure 25:**
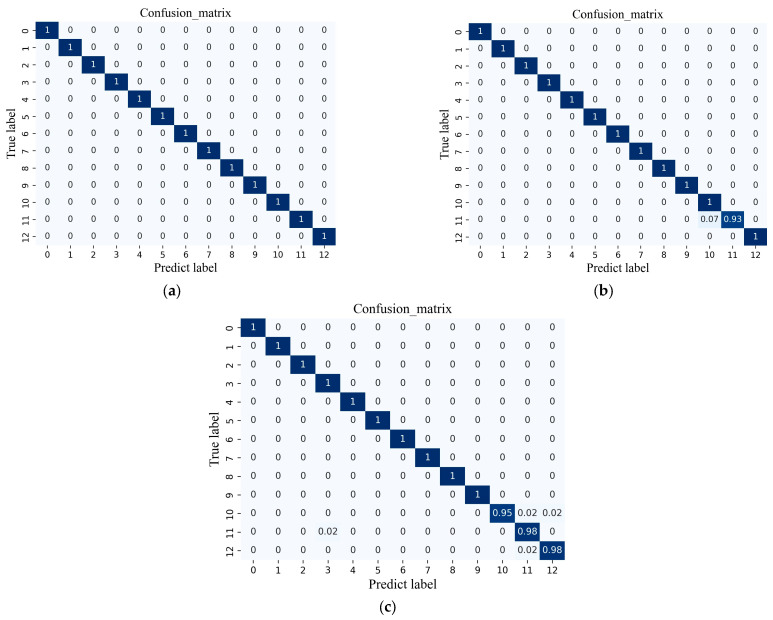
Confusion matrix of the proposed method. (**a**) Dataset A; (**b**) Dataset B; (**c**) Dataset C.

To further demonstrate the fault diagnostic classification outcomes of the proposed NHVT combined with multi-source information fusion, the confusion matrix is offered to gain insights into its strengths and weaknesses, as illustrated in Figure 25.

For 13 different health conditions of mechanical equipment, the proposed method has an observed diagnostic accuracy of more than 90%. It can be observed that the proposed method has a diagnostic accuracy of over 90% for 13 health states of the mechanical equipment. As shown in Figure 25a, all health states of the NHVT can achieve 100%. Meanwhile, the lowest fault diagnosis performance of Label 11 is 93% in Dataset B, as depicted in Figure 25b. Finally, the minimum diagnosis effect of Label 10 in Dataset C is 95%, as described in Figure 25c. The confusion matrix is beneficial for understanding the diagnostic impact of the proposed NHVT and identifying the types of errors made by this method. By analyzing the confusion matrix, we can determine the weaknesses of the NHVT and then make targeted improvements and optimizations.

**Figure 26:**
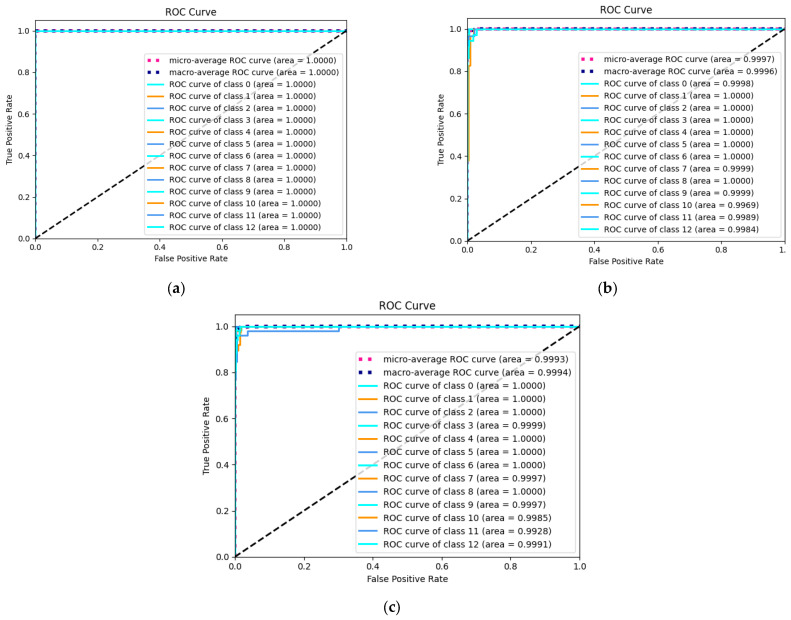
ROC results of the proposed method. (**a**) Dataset A; (**b**) Dataset B; (**c**) Dataset C.

Finally, the ROC curves for the three datasets are charts that display the performance of the NHVT on all fault type thresholds, as shown in Figure 26. The ROC curves for Dataset A show a micro-average value of 100% and a macro-average value of 100%. The ROC curves for Dataset B show the micro-average and macro-average values of 99.97% and 99.96%, respectively. The ROC curves for Dataset C show the micro-average and macro-average values of 99.93% and 99.94%, respectively. Measured by the extent of the ROC curve in Dataset C, health states 3, 7, 9, 10, 11, and 12 diagnosed by the proposed method show weak performance, and the other health states reach 100% of the ROC curve area.

## 5. Discussion

### 5.1. Training Epoch Time

**Figure 27:**
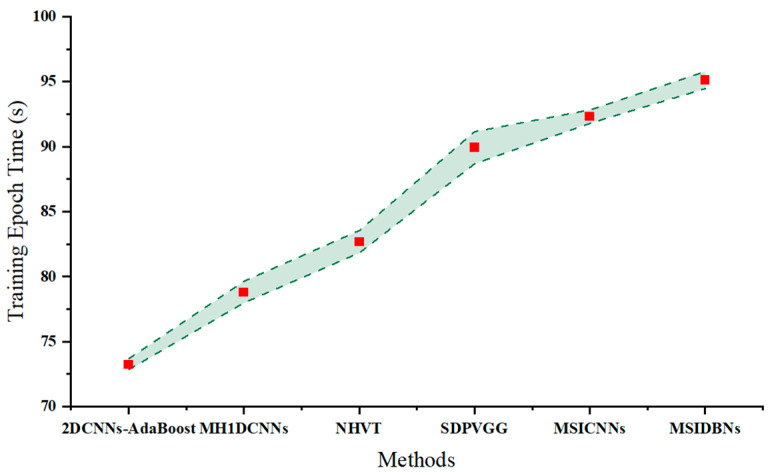
Training epoch time of different methods.

Figure 27 depicts a training time chart that compares the epoch training time of the proposed NHVT to those of the SOTA methods (i.e., SDPVGG, MH1DCNNs, 2DCNNs-AdaBoost, MSICNNs, and MSIDBNs) using Dataset A from case study II.

### 5.2. The Influence of the Training Dataset

**Figure 28:**
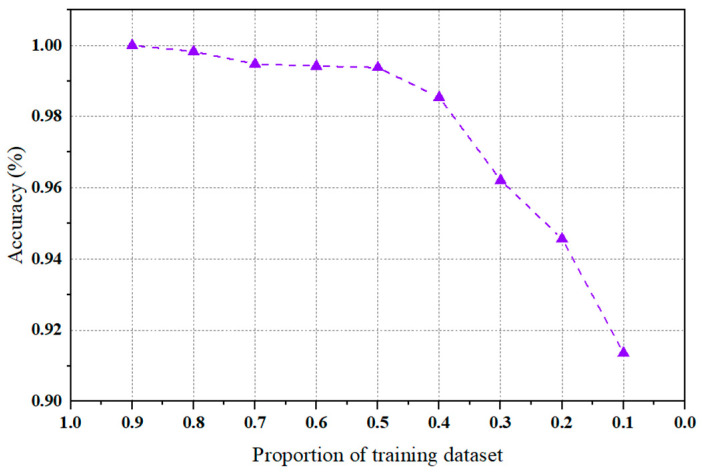
Accuracy of the proposed NHVT based on different proportions of the training dataset.

For further illustration of the tolerance of the proposed NHVT towards different training dataset proportions, we set the training dataset proportion from 0.1 to 0.9; the diagnosis performance diagram is depicted in Figure 28.

As shown in Figure 28, we can conclude that as the proportion of the training dataset decreases, the diagnostic performance of the proposed method continues to decline. Specifically, there is a slight fluctuation in diagnostic accuracy when the ratio drops from 0.9 to 0.5. Even when faced with the most challenging tasks (i.e., the proportion of the training dataset is 0.1), the diagnostic accuracy of this method can still reach 91.36%. Thus, it can be demonstrated that the NHVT can fully assess and utilize the complementary and rich fault features of the multi-source information to obtain more comprehensive abstractions.

### 5.3. The Performance of NHVT under Heavy Noise in Case Study II

**Figure 29:**
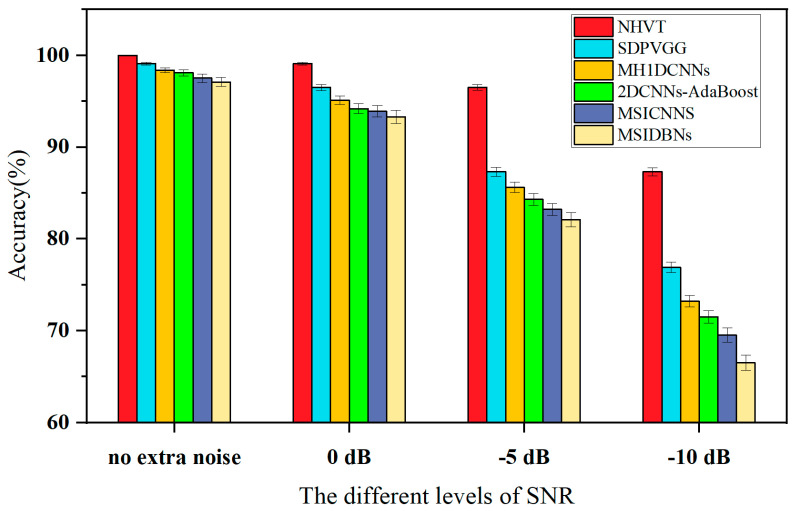
Accuracy of different methods based on various SNRs.

To further investigate the viability and validity of the proposed NHVT under different signal-to-noise ratio conditions, we decided to add Gaussian white noise with signal-to-noise ratios of 0 dB, −5 dB, and −10 dB. With the SNR increases, fault information is more likely to be fluctuated by strong noise. Therefore, it is difficult for all methods to extract effective features from noisy signals under strong interference. The diagnostic results of multi-source information fusion methods (i.e., SDPVGG, MH1DCNNs, 2DCNNs-AdaBoost, MSICNNs, and MSIDBNs) are shown in Figure 29.

Through the diagnostic results, it can be seen that the diagnostic performance of all methods decreases with the increase in signal-to-noise ratio. In detail, the diagnostic accuracy and standard deviation of the NHVT is 99.1% and ±0.15 when the signal-to-noise ratio is 0 dB. And then, the diagnostic accuracy and standard deviation of the proposed method are 96.5% and ±0.32 when the signal-to-noise ratio is −5 dB. Compared with other methods, the diagnostic performance fluctuation of the proposed NHVT is more minor under noise interference, which indicates that the influence of Gaussian white noise on the NHVT is limited. Especially when facing the most demanding working conditions, the diagnostic accuracy of all compared methods is below 80%, but the proposed method still has a diagnostic performance of 89%. It is worth noting that the performance degradation of the proposed method is significantly smaller than for other methods, mainly because it can extract context information of fault signals to extract compelling features under substantial interference.

## 6. Conclusions

In this paper, we introduce a multi-source information fusion framework for the proposed NHVT to overcome the existing drawbacks of insufficient extraction of contextual features and failure information from a single-source signal to realize better fault diagnosis of the mechanical equipment. First, the multi-source information is transformed into time–frequency images to enrich spatial–temporal abstractions by the WT technique. Then, the SwinTransformer and data-level fusion strategy are introduced to fully improve data utilization and diagnostic accuracy. Finally, two case studies display the superiority of the proposed NHVT compared with the SOTA methods.

First, we will develop an online monitoring platform. Then, we will initially train the proposed method with the existing offline data. After that, we will use the proposed NHVT for online monitoring on the online monitoring platform. Most importantly, we will introduce online learning into the proposed framework to endow it with flexibility and generality. In future work, further exploration is needed to alleviate the information redundancy associated with information fusion. In addition, mechanical equipment often operates under variable working conditions, so it is necessary to introduce transfer learning (TL). Finally, we hope to apply experimental theories to practical environments.

## Figures and Tables

**Table 1:** Descriptions of the Paderborn multi-source information dataset.

Speed	Load	Force	Health States		Tasks (Training/Testing Datasets)			Label
			Fault Types	Location	Dataset A	Dataset B	Dataset C	
1500 Rpm	0.7 Nm	1000 N	Electrical discharge trenches	Inner Race	450/50	350/150	250/250	0
			Electrical discharge trenches	Outer Race	450/50	350/150	250/250	1
			Fatigue pitting	Inner Race	450/50	350/150	250/250	2
			Fatigue pitting	Outer Race	450/50	350/150	250/250	3
			Drilling holes	Outer Race	450/50	350/150	250/250	4
			Electric engraver pitting	Inner Race	450/50	350/150	250/250	5
			Electric engraver pitting	Outer Race	450/50	350/150	250/250	6
			Normal	\	450/50	350/150	250/250	7

**Table 2:** Diagnostic accuracy of different methods.

		Tasks (%)		
Setting	Model	Dataset A	Dataset B	Dataset C
Single-source information (only vibration signals)	SAEs	90.82	84.97	63.08
	DBNs	92.38	86.38	65.37
	CNNs	93.84	88.71	68.14
Multi-source information	SDPVGG	97.53	94.28	82.97
	MH1DCNNs	98.15	94.73	83.83
	2DCNN-AdaBoost	98.27	95.62	85.31
	MSICNNs	99.09	96.92	87.50
	MSIDBNs	99.32	97.56	89.77
	NHVT	100	100	100

**Table 3:** The details of the Paderborn bearing dataset.

Shaft Speed (Rpm)	Load (N)	Health States		Tasks (Training/Testing Datasets)			Label
		Fault Location	Fault Width	Dataset A	Dataset B	Dataset C	
2050	200	Inner Race	0.43	1170/130	910/390	650/650	0
		Inner Race	1.01	1170/130	910/390	650/650	1
		Inner Race	1.56	1170/130	910/390	650/650	2
		Inner Race	2.03	1170/130	910/390	650/650	3
		Outer Race	0.42	1170/130	910/390	650/650	4
		Outer Race	0.86	1170/130	910/390	650/650	5
		Outer Race	1.55	1170/130	910/390	650/650	6
		Outer Race	1.97	1170/130	910/390	650/650	7
		Roller	0.49	1170/130	910/390	650/650	8
		Roller	1.16	1170/130	910/390	650/650	9
		Roller	1.73	1170/130	910/390	650/650	10
		Roller	2.12	1170/130	910/390	650/650	11
		Normal	\	1170/130	910/390	650/650	12

**Table 4:** Average diagnosis results of different methods.

Setting	Model	Tasks		
		Dataset A	Dataset B	Dataset C
Single-source	SAEs	90.52% ± 0.712	81.08% ± 0.957	60.79% ± 1.208
	DBNs	91.48% ± 0.664	83.62% ± 0.827	61.31% ± 0.993
	CNNs	93.58% ± 0.528	85.54% ± 0.715	65.12% ± 0.953
Multi-source	SDPVGG	96.36% ± 0.304	91.95% ± 0.394	90.26% ± 0.428
	MH1DCNNs	97.26% ± 0.266	93.27% ± 0.375	91.57% ± 0.387
	2DCNN-AdaBoost	98.53% ± 0.258	95.23% ± 0.364	92.54% ± 0.401
	MSICNNs	99.18% ± 0.241	96.81% ± 0.327	93.81% ± 0.322
	MSIDBNs	99.79% ± 0.197	97.43% ± 0.274	94.72% ± 0.318
	NHVT	100%	99.48% ± 0.213	99.38% ± 0.259

## Data Availability

Data are contained within the article.
